# *Physcomitrella patens* Activates Defense Responses against the Pathogen *Colletotrichum gloeosporioides*

**DOI:** 10.3390/ijms160922280

**Published:** 2015-09-15

**Authors:** Guillermo Reboledo, Raquel del Campo, Alfonso Alvarez, Marcos Montesano, Héctor Mara, Inés Ponce de León

**Affiliations:** 1 Department of Molecular Biology, Clemente Estable Biological Research Institute, Avenida Italia 3318, CP 11600 Montevideo, Uruguay; E-Mails: greboledo@iibce.edu.uy (G.R.); rdelcampo@iibce.edu.uy (R.C.); aalvarez@cin.edu.uy (A.A.); hmara40@hotmail.com (H.M.); 2 Laboratory of Plant Physiology, Nuclear Research Center, Faculty of Sciences, Mataojo 2055, CP 11400 Montevideo, Uruguay; E-Mail: montesano@cin.edu.uy

**Keywords:** *Physcomitrella patens*, *Colletotrichum gloeosporioides*, defense responses, cell wall, chloroplasts relocation, gene expression, auxin signaling

## Abstract

The moss *Physcomitrella patens* is a suitable model plant to analyze the activation of defense mechanisms after pathogen assault. In this study, we show that *Colletotrichum gloeosporioides* isolated from symptomatic citrus fruit infects *P. patens* and cause disease symptoms evidenced by browning and maceration of tissues. After *C. gloeosporioides* infection, *P. patens* reinforces the cell wall by the incorporation of phenolic compounds and induces the expression of a Dirigent-protein-like encoding gene that could lead to the formation of lignin-like polymers. *C. gloeosporioides*-inoculated protonemal cells show cytoplasmic collapse, browning of chloroplasts and modifications of the cell wall. Chloroplasts relocate in cells of infected tissues toward the initially infected *C. gloeosporioides* cells. *P. patens* also induces the expression of the defense genes *PAL* and *CHS* after fungal colonization. *P. patens* reporter lines harboring the auxin-inducible promoter from soybean (GmGH3) fused to β-glucuronidase revealed an auxin response in protonemal tissues, cauloids and leaves of *C. gloeosporioides*-infected moss tissues, indicating the activation of auxin signaling. Thus, *P. patens* is an interesting plant to gain insight into defense mechanisms that have evolved in primitive land plants to cope with microbial pathogens.

## 1. Introduction

Plants respond to pathogen attack by activating the production of a variety of defense-related compounds. After pathogen perception, signaling cascades are activated leading to the increase of reactive oxygen species (ROS), synthesis of antimicrobial compounds, fortification of the cell wall and induction of defense-related genes [1]. The induction of many of these responses is common to both resistant and susceptible hosts, and the outcome of the interaction relies on an effective and rapid host defense response. The hypersensitive response (HR) is a form of cell death that occurs in infected tissues to restrict pathogen growth [2]. Studies in flowering plants have shown that some necrotrophic pathogens need HR cell death to achieve full pathogenicity [3,4]. The biosynthesis of hormones such as salicylic acid, ethylene, jasmonic acid, auxin and abscisic acid is also induced after pathogen recognition and play key roles in plant defense responses [5,6]. Depending on the pathogen, different hormonal pathways are activated leading to resistance to particular pathogens [5]. In addition, some pathogens can produce themselves phytohormones interfering with the plant defense [7].

*Colletotrichum* species are a group of ascomycete plant pathogens that infect and cause anthracnose disease in a wide range of plant species including important crops [8]. Many *Colletotrichum* species are hemibiotrophs having initially a biotrophic phase of infection in living host plant cells and a second destructive necrotrophic mode of infection [9,10]. *Colletotrichum gloeosporioides* (*C. gloeosporioides*) has been associated with at least 470 different host species and is considered as the major causal agent of post-harvest disease in fruits such as citrus, apple, olive, mango, banana and strawberries [11]. On citrus, postharvest anthracnose of fruit is caused by *C. gloeosporioides* (Penz.) Penz. and Sacc. in Penz [12]. *C. gloeosporioides* is a primary invader of injured or weakened tissues leading to un-marketable infected citrus fruits [12]. Infected tissues are usually symptomless and the disease becomes visible, when the peel of the fruit is injured or the fruit is exposed to stress conditions or become senescent [12,13]. Conidia of *C. gloeosporioides* germinate on the surface of the fruit forming melanized appresoria which remains as quiescent infections [13]. When tissues die or are weakened by stress, they are rapidly colonized by *C. gloeosporioides* and acervuli are formed, completing the life cycle [12]. Symptoms of postharvest anthracnose in citrus fruit can vary and lesions can occur around a senescent button before spreading to adjacent rind or form brown to black spots that become sunken on the rind tissues [14]. Post-harvest anthracnose in citrus is increased by ethylene degreening, which is a method used to improve fruit color [12,13,15].

Mosses are bryophytes that are infected with several fungal pathogens which are capable of producing disease in different crops [16]. In mosses, the fungi *Botrytis cinerea*, *Alternaria alternata*, *Fusarium avenaceum* and *Fusarium oxysporum*, and the *oomycetes Pythium irregular* and *Pythium debaryanum*, cause severe necrosis in tissues leading to plant maceration and death [16,17,18,19]. Other fungal pathogens such as *Cladosporium oxysporum* and *Epicoccum nigrum* cause milder discoloration or chlorosis in mosses [16,20]. In response to *B. cinerea*, *P. irregulare* and *P. debaryanum* infection, *P. patens* activate a defense response, evidenced by the fortification of the plant cell wall and induction of defense related genes encoding phenylammonia lyases (PAL) and chalcone synthase (CHS) [18,19]. Other defense mechanisms are activated in *P. patens* in response to *B. cinerea* and elicitors of the phytopathogenic bacteria *Pectobacterium carotovorum* sp. *carotovorum* (*P.c. carotovorum*), including the activation of an HR-like response, the induction of genes encoding oxylipin producing lipoxygenases, and the accumulation of oxylipins derived from an alpha-dioxygenase [17,18,19,21]. In addition, it was recently shown that the auxin signaling pathway is activated in *P. patens* after *P. irregulare* and *P. debaryanum* infection [22]. In the present work we analyzed if an isolate of *C. gloeosporioides* obtained from symptomatic citrus fruits was capable of infecting *P. patens* tissues and activating a defense response. Since we have observed that most necrotophic pathogens cause cell wall fortification and induce the expression of defense genes encoding PAL and CHS, we analyzed these responses, and included the available GH3::GUS reporter line to analyze auxin signaling in response to this pathogen. We show that *C. gloeosporioides* infects *P. patens* cells and cause disease symptoms evidenced by browning of moss tissues and maceration. In response to pathogen infection *P. patens* reinforces the cell wall, changes chloroplast distribution in the infected and the neighbor cells, induces the expression of defense related genes and activates auxin signaling.

## 2. Results and Discussion

### 2.1. Colletotrichum gloeosporioides Isolated from Orange Fruits Infects P. patens and Causes Disease Symptoms

**Figure 1:**
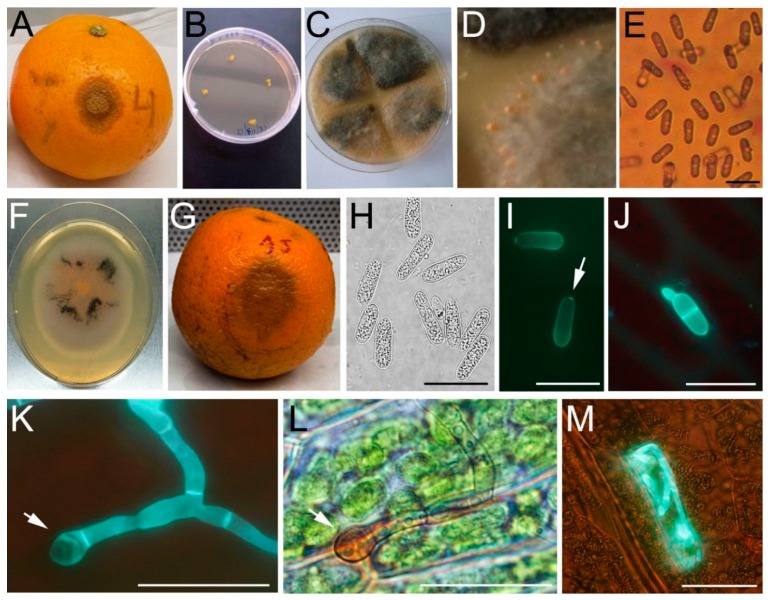
Isolation of *C. gloeosporioides* strain and infection of *P. patens* tissues. (**A**) Symptomatic citrus fruit; (**B**) Small pieces of symptomatic citrus lesion inoculated on Potato Dextrose Agar (PDA); (**C**) Mycelium grown from infected citrus lesion; (**D**) A closer view of C showing the acervuli; (**E**) Conidial suspension obtained from the orange mucilage; (**F**) Colony from monosporic conidial suspension; (**G**) Artificially inoculated citrus fruit; (**H**,**I**) *C. gloeosporioides* conidia morphology. Abscission scar of conidia is indicated with a white arrow in **I**; (**J**) Germinated conidia on leaf surface showing germ tube and septa; (**K**) *C. gloeosporioides* hyphae growing on leaf surface stained with solophenyl; (**L**) Same picture as **K** showing brown mature appresorium. Appresorium is indicated with a white arrow in **K** and **L**; (**M**) Intracellular growth of hyphae in moss leaf cell. Scale bars represent 20 μm in **E**, **H**–**L** and 50 μm in **M**.

A strain of *Colletotrichum* spp., which was genotypically identified as *C. gloeosporioides*, was obtained from a citrus fruit naturally infected, and with dark dry brown lesions that become sunken on the rind tissues (Figure 1A). Small pieces of the citrus lesions were inoculated on the medium leading to the formation of colonies with abundant grey mycelium (Figure 1B,C). The asexual spore-bearing structure known as acervuli produced orange to pink masses of conidia (Figure 1D,E). A monosporic culture was obtained (Figure 1F) and a monosporic conidial suspension was artificially inoculated in citrus fruit developing a brown lesion with sunken parts after 7 days of inoculation (Figure 1G). The isolate recovered from the artificially symptomatic tissue were morphologically identical to the original isolate obtained from the naturally infected fruit, fulfilling Koch’s postulates (Figure 1H,E). The *C. gloeosporioides* strain produced typically cylindrical aseptate conidia with rounded ends (Figure 1H) [23], showing at one of the ends an abscission scar (Figure 1I). In order to analyze if *C. gloeosporioides* infects *P. patens*, moss colonies were inoculated with a conidial suspension and microscopic analysis were conducted. Four hours after inoculation (HAI), several conidia germinated, developed a septa, and formed a germ tube on the surface of moss leaves (Figure 1J). This suggests that like in flowering plants, conidia germination and formation of infection structures in *P. patens*, require contact with a hard surface and probably host signals [24]. *C. gloeosporioides* fungal proteins involved in cytokinesis, polarized cell division and differentiation of the germ tube to appresorium formation have been identified [25,26].

At 24 HAI germ tubes ramified and melanized appresoria were distinguished on the leaves surfaces (Figure 1K–L), and at 48 HAI several moss cells were infected evidenced by fungal hyphae growing inside the cells (Figure 1M). Melanin impregnation of the appressoria wall enables *Colletotrichum* species to build up a high osmotic pressure and penetrate the cell wall [27]. Under our experimental conditions, we did not observe intracellular vesicles associated to the transient biotrophic phase that precedes tissue colonization in a necrotrophic phase of infection [27]. However, in several interactions of *Colletotrichum* species with different hosts, the biotrophic stage is absent [28]. Like in flowering plants, adhesion of *C. gloeosporioides* conidia and appressoria occurred preferentially in the juxtapositions of the cells (Figure 1I–K), which may be favorable for the development of the pathogen, and suggest a direct process of fungal penetration [29,30]. Direct infection is common among various *Colletotrichum* species [31], and involves a combination of mechanical force and the degradation of the cell wall by enzymes [32,33]. Stomata and stomatal subsidiary cells are also penetration sites for *C. gloeosporioides* in some hosts [29,34]. In moss tissues, direct penetration and colonization of wounded tissues is probably the form of invasion by this fungal pathogen since *P. patens* does not have stomata. To the best of our knowledge, this is the first time that a *Colletotrichum* species is reported to infect a moss.

**Figure 2:**
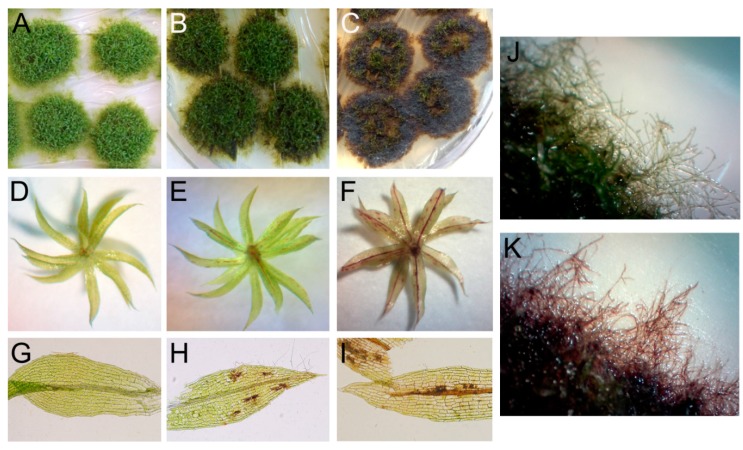
Symptom development in *C. gloeosporioides* inoculated *P. patens* tissues. (**A**) Control moss colonies; (**B**) *C. gloeosporioides* infected moss colonies at 2 days after inoculation (DAI); (**C**) *C. gloeosporioides* infected moss colonies at 5 DAI; (**D**) Control gametophore; (**E**) *C. gloeosporioides* infected gametophore at 2 DAI; (**F**) *C. gloeosporioides* infected gametophore at 5 DAI; (**G**) control leaf; (**H**) *C. gloeosporioides* infected leaf at 2 DAI; (**I**) *C. gloeosporioides* infected leaf at 5 DAI; (**J**) Border of a control moss colony; (**K**) Border of a moss colony inoculated with *C. gloeosporioides* at 5 DAI. In **D**, **E** and **F** gametophores were cut at the lower part of the cauloid and placed upside down for proper visualization of symptoms.

*P. patens* has distinct developmental stages, including the protonema, which is a filamentous network of cells, and the radially symmetric gametophore, which is a leafy shoot composed of a nonvascular cauloid (stem-like structure) with leaves and rhizoids [35]. Disease development can be easily visualized microscopically since leaves, rhizoids and protonemal filaments are formed of a monolayer of cells. We evaluated symptom development at 1, 2, and 5 days after inoculation (DAI) with *C. gloeosporioides*. While no clear symptoms were observed in *P. patens* colonies at 1 DAI (data not shown), moss tissues were susceptible to *C. gloeosporioides* infection at 2 DAI, evidenced by the appearance of brown protonemal tissues at the border of the colonies, brown cells and some brown midline veins in leaves (Figure 2B,E,H), while untreated tissues were green (Figure 2A,D.G). After 5 DAI, protonemal tissues were brown and heavily macerated compared to control green tissues (Figure 2C,J,K).

Five day-inoculated leaves had brown midline veins and brown cells in leaves (Figure 2F–I). Browning and necrosis of tissues is a typical symptom caused by *C. gloeosporioides* in flowering plants [15,36]. During the necrotrophic phase of infection by *Colletotrichum* species, the activity of cell wall degrading enzymes such as endopolygalacturonase and pectate lyase increases [28]. Pectate lyases contribute to virulence in *C. gloeosporioides* and mutants in these type of enzymes produce smaller lesions in avocado [37]. Other cell wall degrading enzymes such as cellulase, α-mannosidase, and 1,4-β-xylanase are also virulent factors of *C. gloeosporioides* [38]. Here, we show that *C. gloeosporioides* is able to infect *P. patens* tissues and cause disease symptoms. *P. patens* has only one layer of cells in most of its tissues including leaves, protonemal filaments and rhizoids, which probably facilitates pathogen invasion and tissue maceration.

### 2.2. P. patens Activates Cell Wall-Associated Defenses against C. gloeosporioides

**Figure 3:**
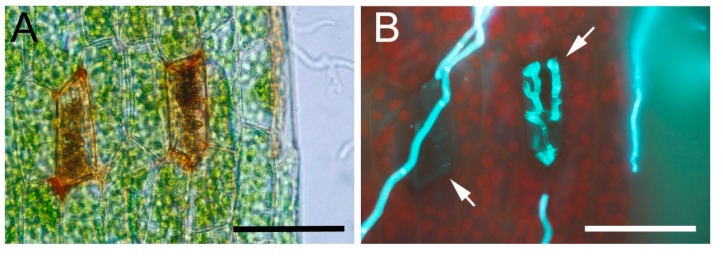

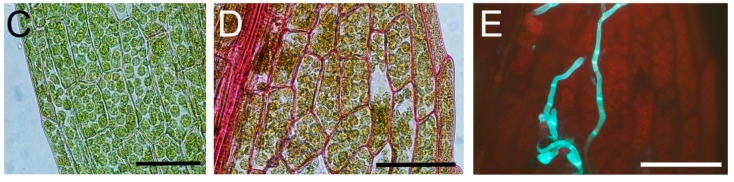
Cell wall-associated defenses in *C. gloeosporioides*-infected tissues. (**A**) *C. gloeosporioides*-infected cells showing brown cell walls at 2 DAI; (**B**) Same leaf as **A** stained with solophenyl, hyphae inside the cells are indicated with a white arrow; (**C**) Control leaf; (**D**) *C. gloeosporioides*-infected leaf stained with safranine-O at 2 DAI; (**E**) Same leaf as **D** stained with solophenyl. Scale bars represent 50 μm.

Cell wall modifications are important defense responses against *Colletotrichum* species since these fungal pathogens often directly penetrate plant cell walls. Consistently, we observed frequently that cell walls of *C. gloeosporioides*-infected cells were brown which could be indicative of phenolic compounds accumulation (Figure 3A,B). We therefore visualized in more detail cell wall-associated defense responses. *C. gloeosporioides*-infected leaves were stained with safranin-O to measure the incorporation of phenolic compounds. *C. gloeosporioides*-infected tissues were positively stained (Figure 3D,E), while untreated leaves (Figure 3C) were not stained, reflecting a cell wall reinforcement mechanism. Similarly, in flowering plants deposition of phenolic compounds has been observed after *C. gloeosporioides* infection [29]. These results indicate that the presence of *C. gloeosporioides* is perceived by *P. patens*, which activates a cell wall fortification mechanism. However, this fortification mechanism is not sufficient to stop fungal colonization leading to plant decay.

### 2.3. Colletotrichum gloeosporioides Infection Causes Cytoplasmic Shrinkage and Chloroplasts Repositioning

**Figure 4:**
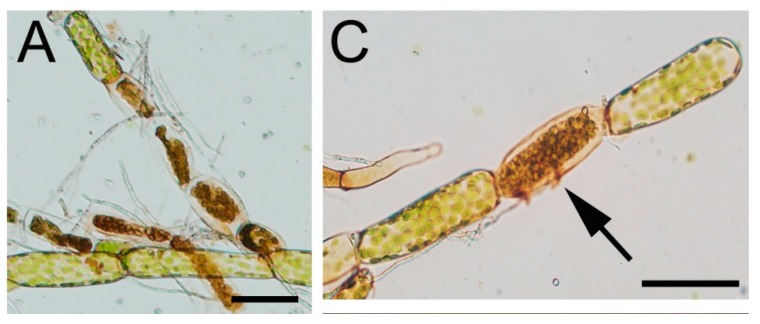

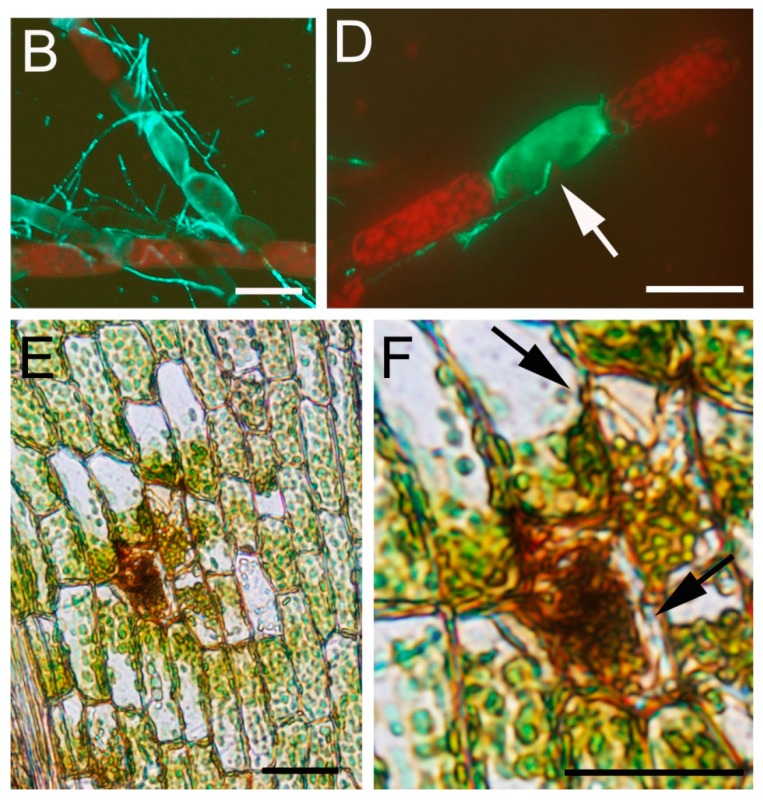
Cytoplasmic shrinkage and intracellular relocation of chloroplasts after *C. gloeo-sporioides* infection. (**A**,**C**) Protonemal tissues inoculated with *C. gloeosporioides*; (**B**,**D**) Protonemal tissues inoculated with *C. gloeosporioides* and stained with solophenyl; (**E**) *C. gloeosporioides* infected leaf showing chloroplast relocation (**F**) Closer view of **E**, hyphae are indicated with black arrows. Arrows in **C** and **D** indicate a hypha in contact with a protonemal cell. Scale bars represent 20 μm in **A**–**D** and 50 μm in **E**–**F**.

Protonemal tissues infected with *C. gloeosporioides* showed cytoplasmic shrinkage, browning of chloroplasts and changes in the cell wall evidenced by staining with the fluorescent dye solophenyl flavine 7GFE 500 (Figure 4A–D). Cytoplasmic shrinkage, browning of the chloroplasts and accumulation of autofluorescent compounds are indicative of an HR-like response. Similar cellular changes occur in *P. patens* tissues infected with HR inducing pathogens such as *B. cinerea* and a *P. carotovorum* strain that have the harpin-encoding gene hrpN [17,19]. An HR is induced in several interactions of *C. gloeosporioides* with flowering plants, including citrus, cowpea and Arabidopsis [13,29,39]. Although in most of these interactions the HR was associated to a resistant mechanism, some susceptible hosts also displayed an HR response [29]. Necrotrophic pathogens are capable of inducing an HR response for their own benefit [4]. Further studies are needed to understand if the HR-like response in *P. patens* facilitates *C. gloeosporioides* infection.

When *C. gloeosporioides*-inoculated leaves were observed in more detail, a relocation of chloroplasts in cells surrounding a brown-infected cell was evident (Figure 4E). Chloroplasts were redistributed during infection in infected cells and in adjacent non-infected cells chloroplasts relocated close to the cell wall and in proximity to the infected cell (Figure 4E,F).

This type of response has also been observed in *P. patens* tissues infected with other pathogens including *P. irregulare*, *P. debaryanum*, *F. avenaceum* and *A. alternata* [18,40]. However, the precise role of chloroplast repositioning towards the infection site is at present unknown. Chloroplasts play important functions in plant defense against pathogens. These organelles are a rich source of ROS, which act as signaling molecules to induce defense gene expression, are involved in cell wall reinforcement, and have antimicrobial activity [1,41]. Treatment of Arabidopsis with flg22, a peptide derived from bacterial flagellins, induces the formation of calcium transients in chloroplasts, which leads to downregulation of photosynthesis-related genes and upregulation of defense genes [42]. Chloroplasts also host biosynthetic pathways for the production of amino acids, defense hormones and secondary metabolites involved in plant resistance against pathogens [43]. Kwon *et al.* [44] have demonstrated that in flowering plants, proteins from intact chloroplast are released to the cytoplasm after *P. carotovorum* infection. In addition, during virus infection, the normally chloroplast localized receptor interacting protein (NRIP1) is recruited to the cytoplasm and nucleus before plant defense is activated [45]. Interestingly, Kaplan *et al.* [46] have demonstrated that in *Arabidopsis* and *Nicotiana*, chloroplasts send out dynamic tubular extensions called stromules during innate immunity, which form numerous connections with the nucleus. These stromules are involved in the transport of pro-defense signals such as H_2_O_2_ into the nucleus during immunity [46]. In *Phytophthora infestans*-potato interaction, the nucleus moves to the pathogen contact site [47]. Further studies are needed to understand the role of chloroplasts repositioning during pathogen infection, including the relation with nucleus movement, and the possible effects of proteins or peptides delivered from chloroplasts on plant defense and pathogens growth.

### 2.4. P. patens Induces Defense Gene Activation in Response to C. gloeosporioides Infection

In order to analyze if *P. patens* activates the expression of defense genes in response to *C. gloeosporioides*, the expression pattern of three defense genes previously known to be induced in this moss in response to pathogen infection was evaluated. The results show that *C. gloeosporioides* infection activates the expression of several defense related genes such as a *PAL*, *CHS* and a gene encoding a dirigent-like protein (*DIR*). All genes were rapidly induced at 4 HAI, which correlates with the germination of conidia and the development of the germination tube. The maximum expression levels of the three genes were reached at 24 HAI. In case of *PAL* and *CHS* two hybridization bands were observed, which correspond to other *PAL* and *CHS* genes with high sequence similarity. *P. patens* has higher number of members forming part of the *PAL* and *CHS* multigene families compared to flowering plants [48,49], and some of the products produced by these enzymes could play a role in the defense response of this moss to pathogens. *P. patens* genome contains 13 additional *PAL* genes, and five of them (Phypa_177179, Phypa_181734, Phypa_178793, Phypa_176961 and Phypa_123073) have higher identity than 80% to the *PAL* gene used as a probe (Phypa_156018; 2877 bp), and encode transcripts varying from 2704 to 3583 bp. Northern blot results could represent the expression pattern of several of these *PAL-encoding* genes. Similarly, *P. patens* has 16 additional *CHS-encoding* genes and eight (Phypa_100508, Phypa_101257, Phypa_110814, Phypa_155379, Phypa_98737, Phypa_152430, Phypa_149682 and Phypa_63283) have higher identity than 80% to the *CHS* gene used as a probe (Phypa_104998; 2191 bp), and encode transcripts varying from 1924 to 2787 bp, which could also represent the expression pattern of some of these CHS-encoding genes observed in the Northern blot analysis. *PAL* and *CHS* defense gene expression also increases in flowering plants infected with *Colletotrichum* species [50,51]. In addition, an increase in PAL activity has been observed in the interaction of *C. gloeosporioides* with cowpea [29], suggesting the involvement of the phenylpropanoid pathway in the defense response against this fungal pathogen. The importance of phenylpropanoid pathway products in the defense response of *Medicago truncatula* to *Colletotrichum trifolii* was confirmed in silenced CHS plants which were more susceptible and showed extensive mycelium development compared to control plants [51]. *C. gloeosporioides* infection also leads to lignification to reinforce the cell wall in flowering plants [29]. DIR proteins mediate the free radical coupling of monolignol plant phenols to yield lignans and lignins [52]. The *P. patens* DIR-like protein used in this study has 31%–40% identity with DIR proteins of flowering plants. *P. patens* has eight additional putative DIR-like genes with identities of 39.7%–96.7% to the *DIR-like* gene (Phypa_110421). As the DIR-like cDNA fragment used as a probe and two other *DIR-like* genes (Phypa_170601 and Phypa_48771) exhibit higher identity than 80% and encode transcripts of similar sizes, Northern blot results could represent the expression pattern of these three DIR-like genes. In *P. patens* DIR expression was also induced in response to *B. cinerea* inoculation [19]. Thus, DIR-like-proteins could lead to the production of lignin-like compounds, with possible roles in defense against pathogens such as *C. gloeosporioides*.

**Figure 5:**
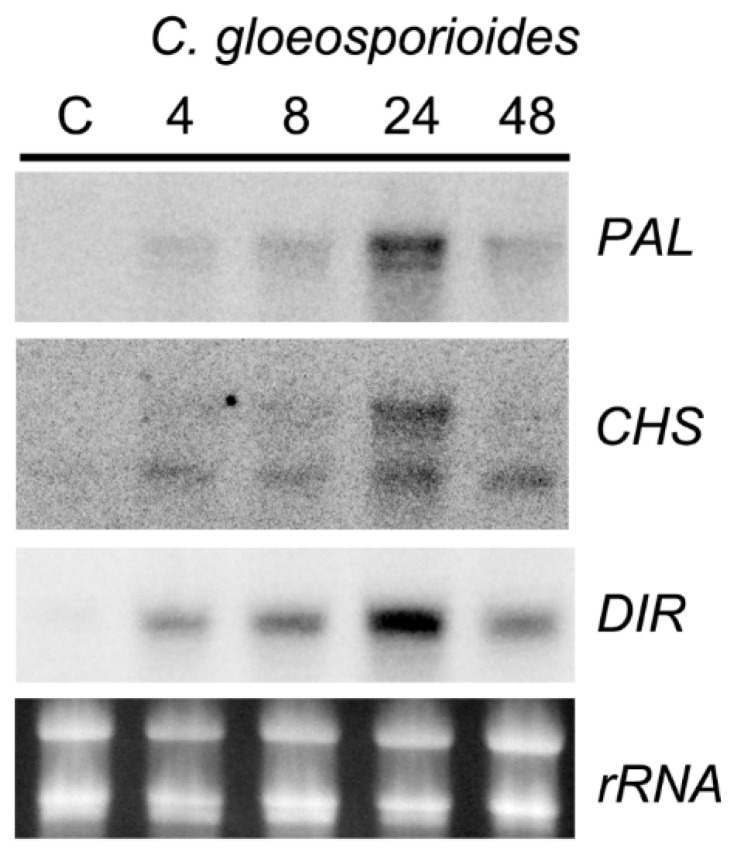
*C. gloeosporioides*-induced expression of defense genes in *P. patens*. Expression of phenylammonia lyase (*PAL*), chalcone synthase (*CHS*) and dirigent-like (*DIR*) genes after *C. gloeosporioides* inoculation. Plants treated during 24 h with water were used as controls. Moss samples were harvested at the indicated times (hours) after treatment. Ten micrograms of total RNA were separated on formaldehyde—agarose gels, transferred to a nylon membrane and hybridized to the corresponding cDNA probes. Ethidium bromide staining of rRNA was used to ensure equal loading of RNA samples. Experiments were repeated twice with similar results.

### 2.5. P. patens Activates Auxin Signaling in Response to C. gloeosporioides Infection

**Figure 6:**
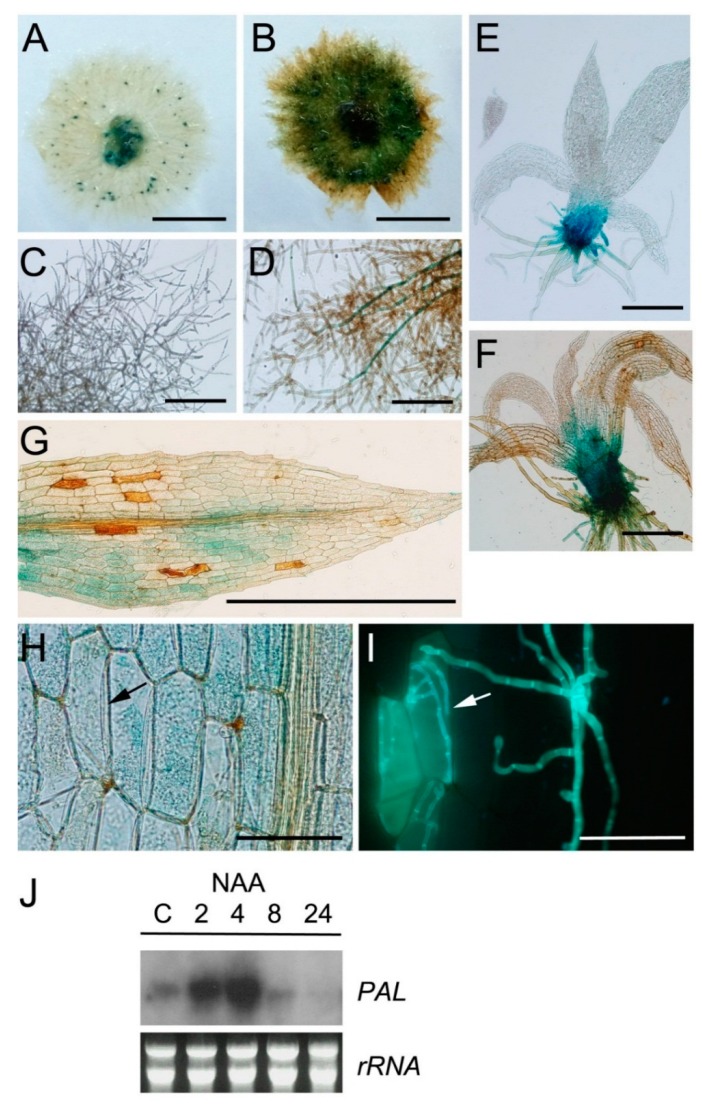
Auxin signaling is activated in *P. patens* after *C. gloeosporioides* inoculation. GUS staining of GH3::GUS reporter line in: (**A**) Untreated colony; (**B**) *C. gloeosporioides*-inoculated colony; (**C**) Untreated protonema; (**D**) *C. gloeosporioides*-inoculated protonema; (**E**) Untreated gametophore; (**F**) *C. gloeosporioides*-inoculated gametophore; (**G**) *C. gloeosporioides*-infected leaf; (**H**) *C. gloeosporioides*-infected leaf; (**I**) Same leaf as H stained with solophenyl. The black arrow in **H** and the white arrow in **I** indicate *C. gloeosporioides*-infected cells. All pictures of *C. gloeosporioides* inoculated tissues were taken at 2 DAI; (**J**) Expression of *PAL* after NAA treatment. Moss samples were harvested at the indicated times (hours) after treatment. Scale bars represent 0.5 cm in **A** and **B**; 0.5 mm in **C**–**G** and 50 μm in **H** and **I**.

*C. gloeosporioides* is able to produce by itself the indole-3-acetic acid (IAA) auxin in culture medium and during infection of flowering plants [53,54,55]. Therefore, we decided to analyze *P. patens* reporter lines harboring an auxin inducible promoter from soybean (GmGH3) fused to β-glucuronidase (GUS) [56], in response to infection with *C. gloeosporioides*. *P. patens* has two GH3 proteins, which are IAA amino acid conjugate synthetases [57]. The results show that while in control tissues GUS staining was detected in spots around the whole colony, tissues inoculated with *C. gloeosporioides* at 2 DAI showed an overall GUS staining except at the border of the colonies (Figure 6A,B). Protonemal tissues are composed of caulonemal filaments involved in substrate colonization and nutrient acquisition, and end chloronemal cells that are principally involved in photosynthesis [58].

Protonemal filaments at the border of the colonies were observed in more detail. Protonemal cells of control colonies did not show GUS accumulation (Figure 6C). In *C. gloeosporioides*-infected protonemal tissues, GUS expression was detected mainly in caulonemal filaments, while chloronemal cells at the border of the colony were not stained (Figure 6D). In control plants GUS was expressed in the basal part of the gametophore (Figure 6E), which is a location of high auxin occurrence [56], leading to the blue spots appearance observed in Figure 6A. When *C. gloeosporioides* infected gametophores were analyzed, the whole cauloid was stained and some GUS stained cells were detectable in leaves (Figure 6F,G). Cells surrounding infected *C. gloeosporioides* cells accumulated GUS (Figure 6H,I). Thus, the results show that auxin signaling is upregulated in *P. patens* tissues infected with *C. gloeosporioides* as evidenced by increased GH3::GUS expression. Since *P. patens* could increase auxin production to induce gene expression needed in the defense response against *C. gloeosporioides*, we analyzed if auxin induced *PAL* expression. The results shown that treatment with auxin (NAA) increased rapidly *PAL* (Phypa_156018) transcript levels in moss tissues (Figure 6J). Interestingly, in citrus flowers, one of the genes most strongly upregulated in response to *Colletotrichum acutatum* is the GH3-encoding gene [59]. GH3::GUS expression also increases in *P. patens* tissues infected with the oomycetes *P. irregulare* and *P. debaryanum* [22]. In addition, *PAL* (Phypa_156018) expression is also induced in *P. irregulare* and *P. debaryanum* infected moss plants [18]. Thus, *P. patens* could induce auxin production and activates auxin signaling to induce defense gene expression. *C. gloeosporioides* produces auxin in the flowering plant *Aeschynomene virginica* [54]. The possible synthesis of auxin by *C. gloeosporioides* in *P. patens* tissues and its effect on moss defense needs further investigation. IAA can inhibit *P. debaryanum* growth *in vitro*, suggesting that the high auxin levels present in the GH3 double knockout plants increase resistance against this pathogen [22]. Auxin signaling is required for resistance against necrotrophs, and Arabidopsis mutant defective in the auxin pathway are more susceptible than wild-type plants to necrotrophic fungus such as *Plectosphaerellacucumerina*, *Botrytis cinerea* and *Alternaria brassicicola* [59,60,61]. Further studies are needed to understand the involvement of auxin homeostasis in moss resistance against *C. gloeosporioides.*

## 3. Experimental Section

### 3.1. Isolation and Identification of Colletotrichum Strain

One isolate of *Colletotrichum* spp. was obtained from lesions formed by natural infections in orange fruit peel tissue from *Citrus reticulata Blanco* “*Ellendale*”*.* The lesions were dry, dark brown and sunken on the rind tissues. Fruits with disease symptoms were obtained from commercial packinghouse in Salto, Uruguay, at the harvest of September, 2013. Isolation was performed by cutting small sections from the border of the anthracnose-like lesions, which were surface sterilized with 1% sodium hypochlorite solution for 3 min, rinsed in sterilized water and dried on a sterile paper in a laminar flow. The tissue segments were placed on Potato Dextrose Agar (PDA) medium in sterilized Petri plates and incubated at 28 °C for seven days. A monosporic culture was made and preserved in 25% glicerol at −80 °C. The *Colletotrichum* spp. isolate was grown on PDA at 28 °C for 10 days to generate spores. Mature and asymptomatic Valencia fruit were surface sterilized with 1% sodium hypochlorite solution for 3 min then rinsing in sterilized distilled water and dried. A spore suspension was adjusted to 1 × 10^5^ spores/mL and immediately 1 mL of the suspension was inoculated with a needle of a syringe between the flavedo and the albedo of three orange fruit (*C. sinensis* (L.) *Osbeck* cv. *Valencia Late*). After inoculation, each fruit was placed in a nylon bag separately, incubated for seven days at 28 °C, with 90%–95% relative humidity and a light/dark cycle of 12/12 h. For DNA extraction, the monosporic isolate was grown on Potato Dextrose Broth at 28 °C during five days, the fungal biomass was filtered, dried at 28 °C for two days and grinded with liquid nitrogen. Fifty milligram were used for extraction with the DNAeasy kit from Qiagen (Hilden, Germany). PCR was performed according to Lima *et al.* [62], using the universal primer ITS4 (5′-TCCTCCGCTTATTGATATGC-3′) coupled with the species-specific primer CgInt for *C. gloeosporioides* (5′-GGCCTCCCGCCTCCGGGCGG-3′).The reaction contained 20 ng of DNA in 50 μL of 10× PCR buffer, 2.5 mM MgCl_2_, 0.2 mM dNTP, 0.5 μM of each oligonucleotides and 0.04 U Taq DNA polymerase (Thermo Scientific, Vilnius, Lithuania). The PCR conditions were as follows: 40 cycles at 94 °C for 1 min, 54 °C for 1 min and 72 °C for 1 min. A PCR fragment with the corresponding size was amplified and sequence analysis confirmed that the *Colletotrichum* isolate corresponds to *C. gloeosporioides* (isolate ICECgS1, Accession number: KT272173).

### 3.2. Physcomitrella patens and Colletotrichum gloeosporioides Growth Conditions

*Physcomitrella patens* Gransden wild-type isolate was grown on agar BCDAT medium. Moss colonies were generated as described previously [18], and grown at 22 °C under a photoperiod of 16 h light. Three-week-old colonies were used for all the experiments. *C. gloeosporioides* was cultivated on 24 g/L PDA (Difco, Houston, TX, USA) at 28 °C.

### 3.3. Colletotrichum gloeosporioides Inoculation and Staining

Conidia were recovered from the orange mucilage of colonies grown on PDA. *C. gloeosporioides* inoculation was performed by spraying a 2 × 10^5^ conidia/mL suspension in water. Symptom development of *C. gloeosporioides*-inoculated *P. patens* colonies was analyzed in three independent experiments using two Petri dishes containing 16 colonies each. *C. gloeosporioides* tissues were stained with 0.1% solophenyl flavine 7GFE 500 in water for 10 min, rinsed in water and visualized with epifluorescence [18]. Photographs were taken at the indicated times after inoculation and representative photographs are shown.

### 3.4. Plant Cell Wall-Associated Defense Responses

Cell wall modifications were detected with safranin-O staining according to Oliver *et al.* [18]. Tissues were incubated with 0.01% safranin-O in 50% ethanol for 5 min. Bright field microscopy and fluorescence microscopy were performed with an Olympus BX61 microscope (Shinjuku-ku, Tokyo, Japan), and all images shown in this study were captured with the Cell F or MICROSUITE software package (Olympus, Tokyo, Japan).

### 3.5. RNA Gel Blot Analysis

Total RNA was isolated from water-treated and *C. gloeosporioides*-inoculated moss tissues using standard procedures based on phenol–chloroform extraction followed by LiCl precipitation. Each sample consisted of 48 colonies. Ten micrograms of total RNA were separated, transferred to nylon membranes, hybridized and washed as described previously [17]. A partial cDNA clone with high level of similarity to DIR-encoding genes from flowering plants [19], a *PAL* and a *CHS* cDNA [63], were amplified by PCR using universal primers or digested with restriction enzymes. Purified fragments were labelled with [α^32^P]-dCTP using the Rediprime II Random Prime labelling system (GE Healthcare, Buckinghamshire, UK). The amount of RNA loaded was verified by the addition of ethidium bromide to the samples and photography under UV light after electrophoresis. Integrity and equal transfer of RNAs to the nylon membranes were confirmed by briefly visualizing the membranes under UV. The blots shown are representative examples of the results obtained in two independent experiments.

### 3.6. GUS Staining

*In situ* localization of GUS activity was performed according to Peleman *et al.* [64]. Tissues were stained at 37 °C for 24 h before destaining in an increasing serial dilution of ethanol, mounted in water, visualized in an Olympus BX61 microscope (Shinjuku-ku, Tokyo, Japan), and images were captured with the Cell F software package (Olympus).

### 3.7. NAA Treatment

*P. patens* colonies were grown for 3 weeks on BCDAT medium and then transferred to medium supplemented with naphthaleneacetic acid (NAA) at a final concentrations of 10 μM.

## 4. Conclusions

In this study, we show that *P. patens* is an interesting plant to gain insight into cellular and molecular mechanisms involved in plant defense responses against pathogens like *C. gloeosporioides* that causes disease in important crops. Further studies can be performed in *P. patens* to identify plant genes involved in the defense response against *C. gloeosporioides* by targeted gene disruption [65]. During recent years, studies focused on moss metabolism have revealed significant differences between *P. patens* and flowering plants, including the absence of the hormone jasmonic acid in *P. patens* [20]. The phenylpropanoid and the auxin pathways represent defense mechanisms against pathogens already present in primitive land plants like mosses. Further studies are needed to understand the phenylpropanoid pathway in *P. patens*, including the identification of the different enzymes and the compounds produced.

## References

[1] Ponce de León I., Montesano M. (2013). Activation of defense mechanisms against pathogens in mosses and flowering plants. Int. J. Mol. Sci..

[2] Morel J.B., Dangl J.L. (1997). The hypersensitive response and the induction of cell death in plants. Cell Death Differ..

[3] Dickman M.B., Park Y.K., Oltersdorf T., Li W., Clemente T., French R. (2001). Abrogation of disease development in plants expressing animal antiapoptotic genes. Proc. Natl. Acad. Sci. USA.

[4] Govrin E.M., Levine A. (2000). The hypersensitive response facilitates plant infection by the necrotrophic pathogen *Botrytis cinerea*. Curr. Biol..

[5] Glazebrook J. (2005). Contrasting mechanisms of defense against biotrophic and necrotrophic pathogens. Annu. Rev. Phytopathol..

[6] Robert-Seilaniantz A., Grant M., Jones J.D. (2011). Hormone crosstalk in plant disease and defense: More than just jasmonate-salicylate antagonism. Annu. Rev. Phytopathol..

[7] Robert-Seilaniantz A., Navarro L., Bari R., Jones J.D. (2007). Pathological hormone imbalances. Curr. Opin. Plant Biol..

[8] Agrios G.N. (2005). Plant Pathology.

[9] O’Connell R.J., Thon M.R., Hacquard S., Amyotte S.G., Kleemann J., Torres M.F., Damm U., Buiate E.A., Epstein L., Alkan N. (2012). Lifestyle transitions in plant pathogenic *Colletotrichum* fungi deciphered by genome and transcriptome analyses. Nat. Genet..

[10] Gan P., Ikeda K., Irieda H., Narusaka M., O’Connell R.J., Narusaka Y., Takano Y., Kubo Y., Shirasu K. (2013). Comparative genomic and transcriptomic analyses reveal the hemibiotrophic stage shift of *Colletotrichum* fungi. New Phytol..

[11] Hyde K.D., Cai L., Cannon P.F., Crouch J.A., Crous P.W., Damm U., Goodwin P.H., Chen H., Johnston P.R., Jones E.B.G. (2009). *Colletotrichum*-names in current use. Fungal Divers..

[12] Timmer L.W., Brown G.E., Sitko S.E. (1998). The role of *Colletotrichum* spp. in postharvest anthracnose of citrus and survival of *C. acutatum* on fruit. Plant Dis..

[13] Brown G.E. (1975). Factors affecting postharvest development of *Colletotrichum gloeosporioides* in citrus fruits. Phytopathology.

[14] Taverner P. (2003). Anthracnose. Packer Newslett..

[15] Brown G.E., Barmore C.R. (1977). The effect of ethylene on susceptibility of Robinson tangerines to anthracnose. Phytopathology.

[16] Akita M., Lehtonen M.T., Koponen H., Marttinen E.M., Valkonen J.P. (2011). Infection of the Sunagoke moss panels with fungal pathogens hampers sustainable greening in urban environments. Sci. Total Environ..

[17] Ponce de León I., Oliver J.P., Castro A., Gaggero C., Bentancor M., Vidal S. (2007). *Erwinia carotovora* elicitors and *Botrytis cinerea* activate defense responses in *Physcomitrella patens*. BMC Plant Biol..

[18] Oliver J.P., Castro A., Gaggero C., Cascón T., Schmelz E.A., Castresana C., Ponce de León I. (2009). *Pythium* infection activates conserved plant defense responses in mosses. Planta.

[19] Ponce de León I., Schmelz E.A., Gaggero C., Castro A., Alvarez A., Montesano M. (2012). *Physcomitrella patens* activates reinforcement of the cell wall, programmed cell death and accumulation of evolutionary conserved defence signals, such as salicylic acid and 12-oxophytodienoic acid, but not jasmonic acid, upon *Botrytis cinerea* infection. Mol. Plant Pathol..

[20] Lehtonen M.T., Akita M., Frank W., Reski R., Valkonen J.P. (2012). Involvement of a class III peroxidase and the mitochondrial protein TSPO in oxidative burst upon treatment of moss plants with a fungal elicitor. Mol. Plant Microbe Interact..

[21] Machado L., Castro A., Hamberg M., Bannenberg G., Gaggero C., Castresana C., Ponce de León I. (2015). The *Physcomitrella patens* unique alpha-dioxygenase participates in both developmental processes and defense responses. BMC Plant Biol..

[22] Mittag J., Šola I., Rusak G., Ludwig-Müller J. (2015). *Physcomitrella patens* auxin conjugate synthetase (GH3) double knockout mutants are more resistant to *Pythium* infection than wild type. J. Plant Physiol..

[23] Agostini J.P., Timmer L.W., Mitchell D.J. (1992). Morphological and pathological characteristics of strains of *Colletotrichum gloeosporioides* from citrus. Phytopathology.

[24] Flaishman M.A., Hwang C.H., Kolattukudy P.E. (1995). Involvement of protein phosphorylation in the induction of appressorium formation in *Colletotrichum gloeosporioides* by its host surface wax and ethylene. Physiol. Mol. Plant Pathol..

[25] Kim Y.K., Kawano T., Li D., Kolattukudy P.E. (2000). A mitogen-activated protein kinase kinase required for induction of cytokinesis and appressorium formation by host signals in the conidia of *Colletotrichum gloeosporioides*. Plant Cell.

[26] Cai Z., Li G., Lin C., Shi T., Zhai L., Chen Y., Huang G. (2013). Identifying pathogenicity genes in the rubber tree anthracnose fungus *Colletotrichum gloeosporioides* through random insertional mutagenesis. Microbiol. Res..

[27] Mendgen K., Struck C., Voegele R.T., Hahn M. (2000). Biotrophy and rust haustoria. Physiol. Mol. Plant Pathol..

[28] Perfect S.E., Hughes H.B., O’Connell R.J., Green J.R. (1999). *Colletotrichum*: A model genus for studies on pathology and fungal-plant interactions. Fungal Genet. Biol..

[29] Barreto A.L.H., Vasconcelos I.M., Grangeiro T.B., Melo V.M.M., Matos T.E., Eloy Y.R.G., Fernandes C.F., Torres D.C., Freire-Filho F.R., Freire F.C.O. (2007). Infection process and host defense responses in compatible and incompatible interactions between cowpea (*Vigna unguiculata*) and *Colletotrichum gloeosporioides*. Int. J. Plant Sci..

[30] Rockenbach M.F., Boneti J.I., Cangahuala-Inocente G.C., Gavioli-Nascimento M.C.A., Guerra M.P. (2015). Histological and proteomics analysis of apple defense responses to the development of *Colletotrichum gloeosporioides* on leaves. Physiol. Mol. Plant Pathol..

[31] Wharton P.S., Uribeondo J.D. (2004). The biology of *Colletotrichum acutatum*. Anales Jard Bot. Madrid.

[32] O’Connell R., Perfect S., Hughes B., Carzaniga R., Bailey J., Green J., Prusky D., Freeman S., Dickman M.B. (2000). Dissecting the cell biology of *Colletotrichum* infection processes. Colletotrichum: Host Specificity, Pathology and Host-Pathogen Interaction.

[33] Latunde-Dada A.O. (2001). *Colletotrichum*: Tales of forcible entry, stealth, transient confinement and breakout. Mol. Plant Pathol..

[34] Ogle H.J., Gowanlock D.H., Irwin J.A.G. (1990). Infection of *Stylosanthes guianensis* and *S. scabra* by *Colletotrichum gloeosporioides*. Phytopathology.

[35] Reski R. (1998). *Physcomitrella* and *Arabidopsis*: The David and Goliath of reverse genetics. Trends Plant Sci..

[36] Kim K.H., Yoon J.B., Park H.G., Park E.W., Kim Y.H. (2004). Structural modifications and programmed cell death of chili pepper fruit related to resistance responses to *Colletotrichum gloeosporioides* infection. Phytopathology.

[37] Yakoby N., Kobiler I., Dinoor A., Prusky D. (2000). pH regulation of pectate lyase secreation modulates the attack of *Colletotrichum gloeosporioides* on avocado fruits. Appl. Environ. Microbiol..

[38] Alkan N., Friedlander G., Ment D., Prusky D., Fluhr R. (2015). Simultaneous transcriptome analysis of *Colletotrichum gloeosporioides* and tomato fruit pathosystem reveals novel fungal pathogenicity and fruit defense strategies. New Phytol..

[39] Hiruma K., Fukunaga S., Bednarek P., Pislewska-Bednarek M., Watanabe S., Narusaka Y., Shirasu K., Takano Y. (2013). Glutathione and tryptophan metabolism are required for *Arabidopsis* immunity during the hypersensitive response to hemibiotrophs. Proc. Natl. Acad. Sci. USA.

[40] Lehtonen M.T., Marttinen E.M., Akita M., Valkonen J.P.T. (2012). Fungi infecting cultivated moss can also cause diseases in crop plants. Ann. Appl. Biol..

[41] Torres M.A., Jones J.D., Dangl J.L. (2006). Reactive oxygen species signaling in response to pathogens. Plant Physiol..

[42] Nomura H., Komori T., Uemura S., Kanda Y., Shimotani K., Nakai K., Furuichi T., Takebayashi K., Sugimoto T., Sano S. (2012). Chloroplast-mediated activation of plant immune signalling in *Arabidopsis*. Nat. Commun..

[43] Trotta A., Rahikainen M., Konert G., Finazzi G., Kangasjärvi S. (2014). Signalling crosstalk in light stress and immune reactions in plants. Philos. Trans. R. Soc. Lond. B.

[44] Kwon K.C., Verma D., Jin S., Singh N.D., Daniell H. (2013). Release of proteins from intact chloroplasts induced by reactive oxygen species during biotic and abiotic stress. PLoS ONE.

[45] Caplan J.L., Mamillapalli P., Burch-Smith T.M., Czymmek K., Dinesh-Kumar S.P. (2008). Chloroplastic protein NRIP1 mediates innate immune receptor recognition of a viral effector. Cell.

[46] Caplan J.L., Kumar A.S., Park E., Padmanabhan M.S., Hoban K., Modla S., Czymmek K., Dinesh-Kumar S.P. (2015). Chloroplast dtromules function during innate immunity. Dev. Cell.

[47] Freytag S., Arabatzis N., Hahlbrock K., Schmelzer E. (1994). Reversible cytoplasmic rearrangements precede wall apposition, hypersensitive cell death and defense-related gene activation in potato/*Phytophthora infestans* interactions. Planta.

[48] Wolf L., Rizzini L., Stracke R., Ulm R., Rensing S.A. (2010). The molecular and physiological responses of *Physcomitrella patens* to ultraviolet-B radiation. Plant Physiol..

[49] Koduri P.K., Gordon G.S., Barker E.I., Colpitts C.C., Ashton N.W., Suh D.Y. (2010). Genome-wide analysis of the chalcone synthase superfamily genes of *Physcomitrella patens*. Plant Mol. Biol..

[50] Tavernier V., Cadiou S., Pageau K., Laugé R., Reisdorf-Cren M., Langin T., Masclaux-Daubresse C. (2007). The plant nitrogen mobilization promoted by *Colletotrichum lindemuthianum* in Phaseolus leaves depends on fungus pathogenicity. J. Exp. Bot..

[51] Samac D.A., Peñuela S., Schnurr J.A., Hunt E.N., Foster-Hartnett D., Vandenbosch K.A., Gantt J.S. (2011). Expression of coordinately regulated defence response genes and analysis of their role in disease resistance in *Medicago truncatula*. Mol. Plant Pathol..

[52] Davin L.B., Lewis N.G. (2000). Dirigent proteins and dirigent sites explain the mystery of specificity of radical precursor coupling in lignan and lignin biosynthesis. Plant Physiol..

[53] Robinson M., Riov J., Sharon A. (1998). Indole-3-acetic acid biosynthesis in *Colletotrichum gloeosporioides* f. sp. *aeschynomene*. Appl. Environ. Microbiol..

[54] Maor R., Haskin S., Levi-Kedmi H., Sharon A. (2004). In planta production of indole-3-acetic acid by *Colletotrichum gloeosporioides* f. sp. *aeschynomene*. Appl. Environ. Microbiol..

[55] Reineke G., Heinze B., Schirawski J., Buttner H., Kahmann R., Basse C.W. (2008). Indole-3-acetic acid (IAA) biosynthesis in the smut fungus *Ustilago maydis* and its relevance for increased IAA levels in infected tissue and host tumor formation. Mol. Plant Pathol..

[56] Bierfreund N.M., Reski R., Decker E.L. (2003). Use of an inducible reporter gene system for the analysis of auxin distribution in the moss *Physcomitrella patens*. Plant Cell Rep..

[57] Ludwig-Müller J., Jülke S., Bierfreund N.M., Decker E.L., Reski R. (2009). Moss (*Physcomitrella patens*) GH3 proteins act in auxin homeostasis. New Phytol..

[58] Menand B., Calder G., Dolan L. (2007). Both chloronemal and caulonemal cells expand by tip growth in the moss *Physcomitrella patens*. J. Exp. Bot..

[59] Lahey K.A., Yuan R., Burns J.K., Ueng P.P., Timmer L.W., Chung K.R. (2004). Induction of phytohormones and differential gene expression in citrus flowers infected by the fungus *Colletotrichum acutatum*. Mol. Plant Microb. Interact..

[60] Llorente F., Muskett P., Sánchez-Vallet A., López G., Ramos B., Sánchez-Rodríguez C., Jordá L., Parker J., Molina A. (2008). Repression of the auxin response pathway increases *Arabidopsis* susceptibility to necrotrophic fungi. Mol. Plant..

[61] Qi L., Yan J., Li Y., Jiang H., Sun J., Chen Q., Li H., Chu J., Yan C., Sun X. (2012). *Arabidopsis thaliana* plants differentially modulate auxin biosynthesis and transport during defense responses to the necrotrophic pathogen *Alternaria brassicicola*. New Phytol..

[62] Lima W.G., Spósito M.B., Amorim L., Goncalves F.P., de Filho P.A.M. (2011). *Colletotrichum gloeosporioides*, a new causal agent of citrus post-bloom fruit drop. Eur. J. Plant Pathol..

[63] Nishiyama T., Fujita T., Shin-I T., Seki M., Nishide H., Uchiyama I., Kamiya A., Carninci P., Hayashizaki Y., Shinozaki K. (2003). Comparative genomics of *Physcomitrella patens* gametophytic transcriptome and *Arabidopsis thaliana*: Implication for land plant evolution. Proc. Natl. Acad. Sci. USA.

[64] Peleman J., Boerjan W., Engler G., Seurinck J., Botterman J., Alliote T., van Montagu M., Inzé D. (1989). Strong cellular preference in the expression of a housekeeping gene of *Arabidopsis thaliana* encoding *S*-adenosylmethionine synthetase. Plant Cell.

[65] Schaefer D.G. (2002). A new moss genetics: Targeted mutagenesis in *Physcomitrella patens*. Annu. Rev. Plant Biol..

